# Informed choice and its associated factors among women received immediate postpartum long-acting reversible contraceptives at public hospitals in Sidama Regional State, Ethiopia, 2022

**DOI:** 10.1186/s40834-023-00229-9

**Published:** 2023-05-10

**Authors:** Beniyam Samuel, Berhan Tsegaye, Dubale Dulla, Amdehiwot Aynalem, Eskinder Israel, Meless Gebrie

**Affiliations:** 1 Department of Midwifery, College of Medicine and Health Science, Dilla University, Dilla, Ethiopia; 2 School of Midwifery, College of Medicine and Health Science, Hawassa University, Hawassa, Ethiopia; 3 School of Nursing, College of Medicine and Health Science, Hawassa University, Hawassa, Ethiopia; 4 Department of Reproductive Health, School of Public health, College of Health Sciences and Medicine, Wolaita Sodo University, Wolaita Sodo, Ethiopia

**Keywords:** Informed choice, Long-acting reversible contraceptives, Method information index plus, Sidama Ethiopia

## Abstract

**Introduction:**

It is crucial to ensure the quality of family planning (FP) services through women's informed choice during the provision of long-acting reversible contraceptives. In Ethiopia, previous studies have focused on the quality of family planning services. However, much emphasis was not given to the informed choice of immediate postpartum long-acting reversible contraceptives (LARCs), particularly in the study area. This study determines the mangnitude of informed choice and associated factors among immediate postpartum women who received long-acting reversible contraceptives.

**Method:**

An institution-based cross-sectional study was conducted from July 1 – August 31, 2022, among 373 immediate postpartum women who received long-acting reversible contraceptives at public hospitals in the Sidama regional state, Ethiopia. Women were selected and interviewed using a systematic random sampling technique and via a structured interviewer-administered questionnaire respectively. Data was collected using Kobo Toolbox software and then exported to the Statistical Package for Social science (SPSS) version 25 for analysis. A logistic regression model was used to identify the predictor variables.

**Results:**

The magnitude of informed choice of long-acting reversible contraceptives was 23.5% (95% CI (19.6%–27.7%)). The messages through posters about long-acting reversible contraceptives at the facility (AOR 3.6, 95% CI (1.92–6.79), postpartum family planning counseling during antenatal care (AOR 2.8, 95% CI (1.2–6.4), previous contraceptive use (AOR 3.23, 95% CI (1.12–9.33), and being secondary and higher educated (AOR 2.92, 95%CI (1.27–6.73) and (AOR 5.7, 95% CI (2.267–14.669) respectively were factors significantly associated with informed choice during immediate postpartum family planning service.

**Conclusion and recommendation:**

In the current study, nearly one-fourth of women were informed about LARCs. Socio-demographic factors, prior use of contraception, exposure to posters that have messages about long-acting reversible contraceptives, and postpartum family planning counselling during antenatal care are factors that affect the woman's ability to make an informed choice. There should be immediate PPFP counselling that focuses on a full range of contraceptive method choices to facilitate postpartum women's ability to make informed choices.

## Introduction

The concept of informed choice has been a cornerstone of reproductive rights and a fundamental tent of quality family planning(FP) services. It emphasizes that clients should choose the method that best meets their personal, reproductive, and health needs based on a thorough understanding of their contraceptive options [1].

At the London Summit on Family Planning in 2012, more than 150 political leaders pledged to make contraceptives accessible to 120 million women in the world's poorest nations by 2020 [2].

The sustainable development goal (SDG) aims to ensure universal access to sexual and reproductive healthcare services, including FP, information, and education, by 2030, and it has also put a new focus on the quality of care, which has accelerated efforts to define and develop measures of service quality [3, 4]. The Nairobi Summit, which was held in 2019, celebrated the 25^th^ anniversary of the International Conference on Population and Development (ICPD) and established the aspirational aim of achieving universal access to high-quality, easily accessible, affordable, and safe modern contraceptives [5].

The new international family planning intiatves, FP2030, was just announced and strives to a future in which all women and girls have the right to live healthy lives and make their own informed choices about using contraception [6]. Health and human rights may be at risk if the information on contraceptive methods is restricted and misrepresented and a lack of effectiveness in counseling may result in a "misinformed decision," which could be detrimental to rights-based strategies [7, 8].

A review of demographic health survey (DHS) data from 34 developing countries revealed that 38% of women had unmet needs for modern FP methods [9]. When users of contraceptives stop using a method, the unmet need for FP increases for reasons other than a decreased need for contraception [10].  Another DHS analysis from 15 different countries found that 7 to 27% of prior users of contraception stopped using it for reasons relating to the quality of care they received [11].

The quality of care in FP has been measured using some tools; however, there is currently no universal agreement on established measurements of quality [12]. A crucial component of high-quality FP services is counseling, which helps women understand what to anticipate when using a contraceptive method and how to deal with health issues and side effects that could be the potential which are primary causes of the discontinuation of contraceptive use [13, 14]. The Method Information Index (MII) was created as an indicator of informed choice by the FP 2020 (FP2020) global initiative to measure FP service quality [15].

As a participant in FP2020 and FP2030, the Ethiopian government committed to providing rights-based family planning methods and ensuring access to counseling and informed FP choice, as well as lowering the maternal mortality ratio from 279 to 279 by 2024/2025 and lowering unmet FP needs from 22 to 17% by 2030 [6, 16, 17].

Despite the improvements in FP service use in Ethiopia, the issue of contraceptive discontinuation and the quality of counseling remain alarming concerns. In Ethiopia, the use of modern contraceptives has increased by 37% over the last 15 years, from 8% in 2000 to 41% in 2019 [18]. Despite the considerable advancements in CPR, the unmet need for contraception during ten years did not significantly diminish; it had been reported at 22%. The quality of counseling needs to be addressed. As per the national PMA survey from 2014 to 2018, just 30% of women reported obtaining enough information during counseling, indicating that the quality of family planning counseling is generally poor [19]. According to the Ethiopian Demographic and Health Survey (EDHS) 2016, performance monitoring Action (PMA) 2019, (PMA)2O21, reported that 35%, 32%, and 35% of Ethiopian women discontinue their contraception within 12 months of initiation respectively [20–23]. In line with this,another study conducted in Ethiopia indicated that only 36.2% of women had made the informed contraceptive choice [24]. A study conducted at an antenatal clinic in Gondar, Ethiopia, showed that only 34.8% of patients were counseled on family planning [25]. Another study that was conducted in Ethiopia revealed that there was no significant improvement in the counseling of family planning among women who received modern contraceptives in 2014 and 2018 and resulted in only 29.9 and 33.4% of women received with informed choice respectively [26].

Even though studies focused on the quality of FP services in Ethiopia, they did not assess an informed choice on family planning, and there is also a limited report about the extent to which women's informed choices are ensured, especially on immediate postpartum long-acting reversible contraceptives in the study area. Knowing the level of informed choice is critical for designing and implementing effective interventions to ensure the quality of postpartum FP services and increase an informed choice coverage. Therefore, the purpose of this study was to determine the magnitude of informed choice and it’s associated factorsamong women who received immediate postpartum long-acting reversible contraceptives at public hospitals in Sidama regional state, Ethiopia.

## Methods

### Study design and setting

An institutional-based cross-sectional study was conducted at public hospitals in the Sidama regional state of Ethiopia from July 1 – August 31, 2022. According to the regional health bureau report in 2022, there are one comprehensive tertiary hospital, five general hospitals, 14 primary hospitals, 137 health centers, and 553 health posts. There are 1490 healthcare workers, and of these, 602 are working in the MCH case team, 385 are midwives, and all public hospitals provide immediate long-term reversible contraceptives, but not all health centers.

### Source population

All women who received immediate postpartum long-acting reversible contraceptives at public hospitals in the region.

### Study population

All women who received immediate postpartum long-acting reversible contraceptives during the data collection period at selected public hospitals in the region.

### Inclusion criteria

Immediate postpartum long-acting reversible contraceptive users at selected public hospitals during the data collection period.

### Exclusion criteria

Women who were severely ill during the data collection period.

### Sample size, technique, and procedure

The sample size was calculated using a single proportion formula using the proportion of an informed choice (36.2%), from the Ethiopian demographic health survey.

$$\mathrm n=(Z\alpha/2)2\mathrm P(1-\mathrm P)/\mathrm d^2={(1.96)}^20.362(1-0.362)/0.05^2=3.8416{(0.05)}^2=355$$

where: n—the required Sample size.

P—Proportion of an informed choice (36.2%), from the Ethiopian demographic health survey (EDHS) 2016 [24].

Z—the 95% CI (1.96) and d—the margin of error, 5%.

After adding a 5% of non-response rate total sample size becomes 373.

### Sampling procedure

Firstly, Adare and Bona from the general hospital, Kebado, Yaye, Tulla, Hula, Chuko, and Aleta Wondo from the primary hospital were selected by lottery, and Hawassa University Comprehensive Specialized Hospital (HUCSH) was selected purposively. The previous nearly two-month average of records for a woman who received immediate postpartum LARCs from all selected hospitals was 890. Of these immediate postpartum (IPP) LARCs users, 64, 420, and 406 were served at HUCSH, two general hospitals, and six primary hospitals in the region, respectively. An average of a similar number of postnatal women was assumed to receive LARCs at these public hospitals during the data collection period, and each hospital was given a proportional share of the required sample size based on the number of clients they served in the past two months. Finally, postpartum women who received LARCs were selected using a systematic random sampling technique by calculating the k value. Based on the K value, which is k = (N/n) (890/373 = 2), the first study unit was selected randomly.

### Data collection tool and procedure

A structured English version interviewer-administered questionnaire was adapted from different literature was translated to the local language (Sidamuafoo). The dependent variable was measured by the “method information index plus” which was adopted from the FP2030 which is the recent global initiative [27]. Independent variable instruments adapted and developed different sources [24, 28]. The questionnaire was programmed into Kobo toolbox software for data collection purposes. The data was collected using the kobo collect mobile application from immediate postpartum women who had received LARCs at each selected public hospital. Four BSc midwives with prior experience in data collection were used, and the process was supervised by two professionals with master's degrees in public health who have prior research supervision experience. To limit the possibility of ‘courtesy bias’ (the tendency to provide socially acceptable answers), the interviews were conducted in a manner that provides privacy to the study participants.

### Data quality assurance

The data collection tool was first prepared in English and then translated into Sidaamu afoo. The tools were retranslated back into the original one to evaluate its consistency. Edited final versions of the questionnaires were used to collect data from respondents. The questionnaires were pretested just one weeks prior to the actual data collection using 19 eligible women (5% of the sample size) at hawassa millonium health center from june 23–28, 2022, based on which some modifications were made to the originally prepared tool. Two days of training on the purpose of research, data collection tools, and on how to conduct client exit interview during data collection were given to data collectors. The completeness of surveys was verified and corrected by supervisors and lead investigators, and required comments were given to data collectors.

### Operational definition

#### Method information index plus (MII ( +)

consists of four questions: Were you informed about other methods?, Were you informed about side effects? Were you told what to do if you experienced side effects? Were you told about the possibility of switching to another route if the method you have chosen is not suitable [27]?

### Informed choice of immediate postpartum LARCs

Women who answered “yes” to all four questions of MII ( +) [27].

### Data entry and analysis

The collected data was downloaded from the Kobo toolbox server into an SPSS fileand then analyzed using SPSS version 25. Frequency and percentage were computed. A logistic regression analysis model was used to identify predictor variables. Independent variables with a *P*-value of less than 0.25 during bivariable analysis were selected for multivariable logistic regression analysis. A multivariable logistic regression analysis was then carried out to identify statistically significant variables. Statistical significance was set at a *P* value of less than 0.05 model fitness was checked by Hosmer and Lemeshow's goodness of fit. To assess the possibility of multicollinearity among the independent variables, the variance inflation factor (VIF) was used.

## Results

In this study, a total of 373 women who use LARCs were interviewed, with a response rate of 100%. The mean age of the participants was 26 (SD ± 6.834) years. Of the total participants, 120 (32.2%) were in the age group of 25–29 years (Table 1).

**Table 1:** Socio-demographic and economic characteristics of study participants at public hospitals in Sidama region, Ethiopia, August 2022 (*n* = 373)

Variables	Frequency	Percent(%)
**Age**		
15–19	40	10.7
20–24	94	25.2
25–29	120	32.2
30–34	58	15.5
35–39	43	11.5
40–44	18	4.8
**Residence**		
Urban	109	29.2
Rural	264	70.8
**Religion**		
Orthodox	85	22.8
Muslim	16	4.3
Protestant	237	64.1
Catholic	31	8.3
**Educational status**		
No formal education	172	46.1
Primary	88	23.6
Secondary	69	18.5
Higher	44	11.8
**Occupation**		
Housewife	133	35.7
Professional	98	26.3
Agriculture	103	27.6
Merchant	39	10.5
**Husband’s occupation**		
Not working	17	4.6
Professional	142	38.1
Agriculture	147	39.4
Merchant	62	16.6
Others^a^	5	1.3
**Husband’s education status**		
No formal education	166	44.5
Primary education	84	22.5
Secondary education	74	19.8
Higher	49	13.1
**Income level (ETB**)		
Below 2000	118	31.6
2000–3000	83	22.3
3001–5000	116	31.1
above 5000	56	15.0

*ETB* Ethiopian birr

*Others*^a^ Daily loborers

Less than half (43.4%) of the women had at least one antenatal care (ANC) visit, among those, only 42 (13.33%) received counseling on postpartum FP methods during ANC. More than half, 195 (52.3%) of them, utilized immediate Implanon (Table 2).

**Table 2:** Reproductive history of study participants at public hospitals in Sidama region, Ethiopia, August 2022 (*n* = 373)

Variables	Frequency	Percent(%)	
**Parity**			
1	133		35.7
2–3	126		33.8
≥ 4	114		30.6
**Number of ANC visits**			
0	58		15.5
1–3	162		43.4
≥ 4	153		41.0
**Received postpartum FP counseling during ANC**			
Yes	42		13.33
No	273		88.74
**Delivery mode**			
SVD	347		87.7
Cesarean section (CS)	26		7.3
**Delivery attendant**			
Midwife	327		87.7
Doctor	18		4.8
IESO	28		7.5
**Prior use of contraception**			
Yes	292		78.3
No	81		21.7
**pregnancy intention**			
Wanted	298		79.9
Unwanted	75		20.1
**Contraceptive method received**			
IUD	18		4.8
Implanon	195		52.3
Judelle	160		42.9

zz

*IESO* Integrated Surgery Officer

*SVD* spontaneous vaginal delivery

A total of 47 (12.6%) and 95 (25.5%) people watched television and listened to the radio at least once each week respectively. A majority of women, 341 (91.4%) do not read a newspaper at all (Table 3).

**Table 3:** Source of information of study participants at public hospitals in Sidama region Ethiopia, August 2022 (*n* = 373)

Variables	Frequency	Percent (%)
**Read the newspaper**		
Not at all	341	91.4
Less than once a week	16	4.3
At least once a week	16	4.3
**Listen to the radio**		
Not at all	241	64.6
Less than once a week	37	9.9
At least once a week	95	25.5
**Watch the television**		
Not at all	286	76.7
Less than once a week	40	10.7
At least once a week	47	12.6
**Own a mobile telephone**		
Yes	118	31.6
No	255	68.4
**Do you use the Internet**		
Yes	15	4.0
No	358	96.0
**Heard about LARCs on the radio in the last few months**		
Yes	116	31.1
No	257	68.9
**Heard about LARCs on television in the last months**		
Yes	75	20.1
No	298	79.9
**Read about LARCsat on poster/leaflet**		
Yes	28	7.5
No	345	92.5
**Heard about LARC at the community event/ conversation**		
Yes	165	44.2
No	208	55.8
**Seen anything about LARCs on the Internet**		
Yes	15	4.0
No	358	96.0
**Seen posters advertising LARCs in the facility**		
Yes	143	38.3
No	230	61.7

### The magnitude of informed choice on immediate postpartum LARCs

The overall prevalence of informed choice on immediate postpartum LARCs was 23.5% (95% CI 19.6% –27.7%). Of immediate postpartum LARCs users, 201(53.9%) and 190 (50.9%) were informed about potential side effects and informed about the possibility of switching to another route if the method chosen is not suitable respectively(Table 4).

**Table 4:** Method information index plus of study participants at public hospitals in sidama regional state of Ethiopia, August 2022 (*n* = 373)

Variables	Frequency	Percent
**Informed about side effects might have with the method**		
Yes	201	53.9
No	172	46.1
**Informed about what to do if you experienced side effects**		
Yes	142	38.1
No	231	61.9
**Informed about other options**		
Yes	174	46.6
No	199	53.4
**Informed about the possibility of switching to another route if the method have chosen is not suitable**		
Yes	190	50.9
No	183	49.1
**The magnitude of the informed choice**		
Yes, to all four questions	88	23.5
No, at least one questions	285	76.5

### Factors associated with the informed choice of immediate postpartum LARCs

In bivariable logistic regression analysis, eight variables were included. These are husband’s educational status, women's educational status, woman’s occupation, husband’s occupation, hearing about LARCs at the community conversation, exposure to posters that promote family planning in the facility, receiving counseling about postpartum family planning during ANC visits, previous contraception use, and the frequency of listening to radio was associated with an informed choice of LARCs.

In multivariable logistic regression analysis, women who received counselling about PPFP during ANC visits, saw posters promoting FP in the facility, the educational status of women, and prior use of contraception were significantly associated with an informed choice of immediate post-partum LARCs.

Women who received immediate postpartum LARCs from facilities that had posters with messages on FP were 3.6 times more likely to make informed choices when compared to those who received services from facilities that did not post posters with messages on FP (AOR 3.6, 95% CI (1.922, 6.79)). Women who received PPFP counseling during an ANC visit were 2.8 times more likely to make informed choices when compared to those who did not receive counseling about FP methods (AOR 2.8, 95%CI (1.2, 6.4)). Women who had ever used contraception were 3.2 times more likely than those who had never used contraception to make informed choices (AOR 3.23, 95%CI (1.12–9). Compared to those with no education, those with secondary and higher education were 2.9 and 3.69 times more likely to make an informed choice as compared to those with no formal education (AOR 2.92, 95%CI (1.266, 6.73), and AOR 5.7, CI 95% (2.267, 14.669), respectively (Table 5).

**Table 5:** Factors associated with the informed choice of L ARCs at public hospitals in Sidama region Ethiopia, August 2022 (*n* = 373)

Variable	**Informed choice**		**OR (95% CI)**	
	Yes	No	**COR (95% CI)**	**AOR95% CI)**
**Educational status**				
Primary	14	74	1.7(.807,3.687)	1.115(.460, 2.703)
Secondary	23	46	4.56(2.246,9.254)	2.9(1.266,6.73)*
Higher	19	25	6.93(3.180,15.09)	5.7(2.267,14.669)*
No formal education	17	155	1	1
**Occupation**				
Professional	29	69	2.5(1.315,4.836)	2.2(.994, 4.858)
Agriculture	22	81	1.6(.828, 3.206)	1.57(.702–3.526)
Merchant	3	36	.500 (.140, 1.788)	.68 (.145–3.186)
No formal job	19	114	1	1
**Husband education**				
Primary	14	70	1.2 (.603, 2.653)	1.352(.569–3.216)
Secondary	21	53	2.46(1.260, 4.816)	2.05(.872,4.81)
Higher	15	34	2.7 (1.295, 5.809)	2.08(.852–5.078)
No formal education	23	143	1	1
**PPFP counseling during ANC**				
Yes	17	25	3.34(6.817,6.590)	2.8(1.224,6.4)*
No	17	275	1	1
**Previous contraception use**				
Yes	68	5	4.6(1.794,11.868)	3.2(1.12–9.325)*
No	224	76	1	1
**Frequency of listening to a radio**				
Less than once a week	3	34	.418 (.123–1.425)	.398 (.100–1.581)
At least once a week	28	67	1.98 (1.140, 3.441)	1.95(.998–3.792)
Not at all	42	199	1	1
**Heard about LARCs at community conversation**				
Yes	37	128	1.38(.827–2.306)	1.42(.732–2.750)
No	36	172	1	1
**Seen posters with messages about LARCs in the facility**				
Yes	51	92	5.2(3.003–9.147)	3.6(1.922,6.79)
No	22	208	1	1

*COR* Crude Odds Ratio, *AOR* Adjusted Odds Ratio

^*^p < 0.05

## Discussion

This study aimed to determine the magnitude of informed choice among immediate postpartum women who received LARCs at public hospitals in the Sidama region, Ethiopia. Determining the magnitude of an informed choice and associated factors among the women who received immediate postpartum LARCs is crucial for achieving the global and national goals.

Counseling that is client-centered respects the client's rights to information, unrestricted access to services, and the ability to make an informed choice [29]. FP programs must provide clients with quick access to a variety of methods, thorough and accurate information about these methods, and assistance in considering the options in order to promote people's right to make an informed choice regarding contraceptive methods [30].

In this study, 23.5% of women made an informed choice about immediate postpartum LARCs. This study suggests that many women who used immediate postpartum LARCs were unaware of their side effects and other alternatives. The inability to make informed choices due to this lack of deeper understanding about contraception could lead to service users ceasing to use them. The finding was in line with the study conducted in Bangladesh and the finding from the Indonesian DHS, which reported that 20% and 24.4% of the women made an informed choice, respectively [31, 32]. Nevertheless, it was lower than other studies in India DHS (36.1%) [33], Pakistan DHS (64.6%) [34], Indonesian DHS (29.3% in 2015 and 28.6% in 2017 [32, 35], Uganda (73%) [36], PMA Kenya (56.7%) [37], and Ethiopia (29.9 in 2014 and 32.4% in 2018) [26] and (36.2%) [24]. The possible reasons for this difference could be the study setting, study design, or sociocultural variations.

According to this study, a sizable proportion of women who received immediate postpartum LARC were not told how to manage side effects if they occurred and not informed about alternative methods. The lack of deeper understanding required to make an informed choice may be the result in the unmet need of LARC.This might be the result of poor client-provider interactions and inadequate communication [38], Higher levels of unmet need are also a result of a lack of understanding about other methods of contraception. Unmet needs may arise as a result of barriers to family planning services, a lack of techniques, high costs, and the ignorance of couples who are of reproductive age regarding the various forms of contraception and their side effect. Thus, expanding informed choice coverage is a crucial method to address the growing unmet need.

In this study, several factors were identified that affected choice of LARCs methods among post natal women who received immediate post natal LARC at public hospital sidaama region in Ethiopia. The messages through the posters about LARCs at the facilities, postpartum family planning counseling, and women with secondary and higher education were factors significantly associated with an informed choice of immediate postpartum LARCs.

In this study, women who attended secondary and higher levels of education were more likely to make informed choices than women who did not attend school. This finding is in line with other studies [26, 39]. This might be due to the reason that women with secondary and higher education have more knowledge to deal with healthcare professionals.

This study also revealed that women who had ever used contraception previously had an increased chance of receiving an informed choice of immediate postpartum LARCs than women who had never used it. This result is consistent with other studies [40, 41]. This might be due to the reason that women with a previous history are more familiar with contraceptive methods. On the other hand, this might also suggest that healthcare professionals are reluctant to counsel women who have never utilized FP [42]. However, the immediate postpartum period is an appropriate time to discuss or initiate contraception, especially among first-time users and women in environments with limited resources because these women may have fewer opportunities to return to an institution for additional postnatal care.

The current study has shown that women who received FP counseling during ANC were more likely to make an informed choice of LARCs than those who did not get such counseling. This is consistent with studies that were done in Mexico, Uttar Pradesh India, and Ghana [41, 43, 44]. This could be a result of regular interactions between pregnant women and healthcare providers during ANC provides plenty of opportunities to talk about family planning when couples are not preoccupied with a new baby and have time to discuss all choices.

Women who received immediate postpartum LARCs from facilities that posted posters with messages about LARCs were more likely to make an informed choice of LARCs than women who received services from facilities that did not do so. This is consistent with a previous study that was carried out at Kambata Tambaro in southern Ethiopia [45]. This could be when clients obtain important information about family planning services; they would receive the method that suits them, and it could also prepare them to receive the services.

## Conclusion and recommendation

In the current study, nearly one-fourth of the study participants had been informed about immediate postpartum LARCs. The messages through the posters about LARCs at the facilities, postpartum family planning counseling, and women with secondary and higher education were factors significantly associated with an informed choice of immediate postpartum LARC. Thus, efforts are needed to ensure that all women using immediate postpartum long-acting reversible contraceptives can make an informed choice. The federal, regional, and local stakeholders should place special emphasis on offering a comprehensive integrated package of services, including FP information and counselling with MNCH services like ANC, that can enhance IPPLARC use and the ability to make informed choices. Health care providers should be conscious of any bias that may be present depending on women’s status and counsele all immediate postpartum women. It is better if a future reseachers undertake qualitative study among family planning providers to understand their perspectives, experiences, and challenges in providing modern contraceptives in assuring informed choice.

## Abbreviations

AOR: Adjusted Odds Ratio
CI: Confidence Interval
EDHS: Ethiopian Demographic Health Survey
DHS: Demographic Health Survey
FP: Family planning
HCSTH: Hawassa comprehensive and specialized teaching hospital
IUCD: Intra-Uterine Device
IPPFP: Immediate, Postpartum Family Planning
IPPLARCs: Immediate Postpartum Long Acting Reversible Contraceptives
IRB: Institutional Review Board
IESO: Integrated Surgery Officer
LARC: Long Acting Reversible Contraceptive
MII: Method Information Index
PMA: Performance Monitoring For Action
SVD: Spontaneous vaginal delivery
